# Author Correction: Brains and algorithms partially converge in natural language processing

**DOI:** 10.1038/s42003-023-04776-4

**Published:** 2023-04-11

**Authors:** Charlotte Caucheteux, Jean-Rémi King

**Affiliations:** 1 Facebook AI Research, Paris, France; 2 Université Paris-Saclay, Inria, CEA, Palaiseau, France; 3 École normale supérieure, PSL University, CNRS, Paris, France

**Keywords:** Language, Neural encoding, Network models

Correction to: *Communications Biology* 10.1038/s42003-022-03036-1, published online 16 February 2022.

The original version of this Article contained the wrong file for Supplementary Movie 2. That file has now been replaced with the correct file. The HTML version of this Article has been corrected.

## Supplementary information

Supplementary Movie 2

