# Supplementary material for: Brains and algorithms partially converge in natural language processing
Source: Commun Biol. 2022 Feb 16;5:134. doi: 10.1038/s42003-022-03036-1 (PMC8850612; doi:10.1038/s42003-022-03036-1)
Supplement: Supplementary file 2 — Supplementary Information (new) [file 42003_2022_3036_MOESM2_ESM.pdf]

Supplementary Information

Supplementary Note 1. Average brain responses to reading

When and where do textual sentences elicit brain activity? As expected [4, 3, 6, 7], average fMRI and MEG responses to written words peak in a distributed and bilateral cortical network, including the primary visual cortex, the left fusiform gyrus, the supra-marginal, and the superior temporal cortices, as well as the motor, premotor and infero-frontal areas (Figure 2a). MEG source reconstruction, based on structural MRI and minimum norm estimates, further clarifies the dynamics of this cortical network: on average, word onset elicits a series of brain responses originating in V1 around $\approx$100 ms and continuing within the left posterior fusiform gyrus around 200 ms, the superior and middle temporal gyri, as well as the pre-motor and infero-frontal cortices between 150 and 500 ms after word onset (Figure 2a, Supplementary Movie 1).

Supplementary Note 2. Shared-response model (or noise ceilings)

Shared-response model (SRM) comparison (often referred to as "noise ceiling"), allows us to evaluate the extent to which individual subjects' brain responses can be explained with a model-free approach [1] and can serve as a proxy for a signal-to-noise ratio analysis. For this, we fit, for each subject separately, an SRM model (or noise-ceiling): for each recording of each subject and each sentence Y_{train}, we fit a linear model W from the recordings of all other subjects who read the same sentence X_{train} to predict each voxel and each MEG sensor at each time sample, separately. Using a cross-validation scheme across sentences, we then evaluate the Pearson correlation R between (1) the true brain responses of subject Y_{test} and (2) the predicted brain responses $\hat{Y}_{\text{test}} = W \cdot X_{\text{test}}$ for each voxel and each MEG sensor separately. This procedure can be thought of as approximating an optimal black box: i.e. evaluating a one-hot encoder of brain responses is trained and evaluated on each element of a unique sentence. Noise ceiling peaks within the expected language network [5] (Figure 1f-h). These estimates are relatively low: for example, fMRI noise ceilings reach, on average, $R = 0.129$ (± 0.004 SEM across subjects) in the superior temporal gyrus, whereas MEG noise ceilings peak at $R = 0.069 \pm 0.001$ (Supplementary Table 1).

Supplementary Note 3. Probe analysis of the language transformer

Middle layers better map onto brain responses than input and output layers. Why is there such a difference between layers? To tackle the question, we measure the level to which the 32,400 transformer embeddings linearly predict two types of linguistic features: part-of-speech (i.e a lexical feature), and the number of open and pending nodes (i.e compositional syntactic features [8]). More precisely, we fit and evaluate an ℓ_2-penalized linear model to predict each of these features given the transformer's embedding and plot this decoding performance as a function of the language performance of the model (Figure 2). While the word embedding and middle layers similarly predict word-level features (word length and part-of-speech of the word), the two high-level syntactic features (number of open and pending nodes) are better predicted by the middle layers of transformers. Finally, the decoding performance of the two syntactic features varies with the layer and the performance, in a manner strikingly similar to the brain score. These analyses suggest that middle layers are more brain-like than extremity layers because they learn to encode abstract linguistic properties like syntax.

Supplementary Note 4. Definition of compositionality

Following a recently proposed taxonomy [2], we formally define "compositional" as the language representations that cannot be explained by the linear combination of lexical representations.

This definition may not be fully aligned with the many definitions of compositionality proposed over the years [10]. Specifically, some linguists restrict compositionality to the limited, generally invertible, combinations of words that follow the laws of syntax, and would consequently thus prefer the term "contextual". We believe, however, that the latter term does not clearly point to the representations that are more than the sum of their parts [9] which is critical to the present analyses (Figure 3).

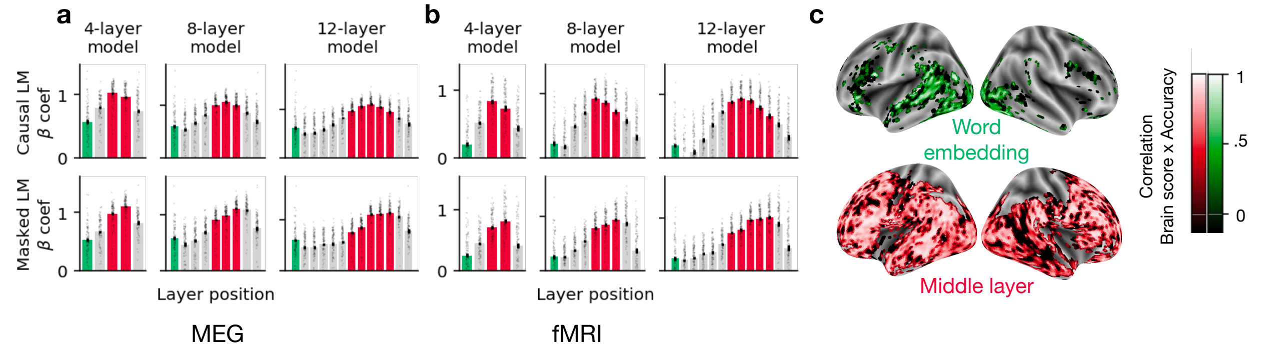

Supplementary Figure 1: **Correlation between the network's performance and brain score.** **a-b.** Standardized beta coefficients between the language modeling performance of the network and its MEG (a) or fMRI (b) scores. For each subject, the brain scores are first scaled (0-mean, 1-std). Then, a linear regression is fit to predict the brain score (averaged across channels and time for MEG, across voxels for fMRI) of each layer of 100 networks (all 512-dimensional, with 12 layers and 8 heads) given their language performance (top-1 accuracy). The beta coefficients of the language performance are reported (y-axis). Results are consistent across 4-, 8-, and 12-layer transformers, trained on a causal (top) or masked (bottom) language modeling task. Error bars are the standard error of the mean beta coefficients across subjects. **c.** Pearson correlation between the performance of the 100 transformers (all 512-dimensional, with 12 layers and 8 heads) and the brain score of their word embedding (top) and ninth layer (bottom), for each voxel. Correlation scores are computed for each (subject, voxel) pair, then averaged across subjects. Only significant voxels are displayed, as assessed with a two-sided Wilcoxon test across subjects and corrected for multiple comparison using false discovery rate across voxels (threshold: .001).

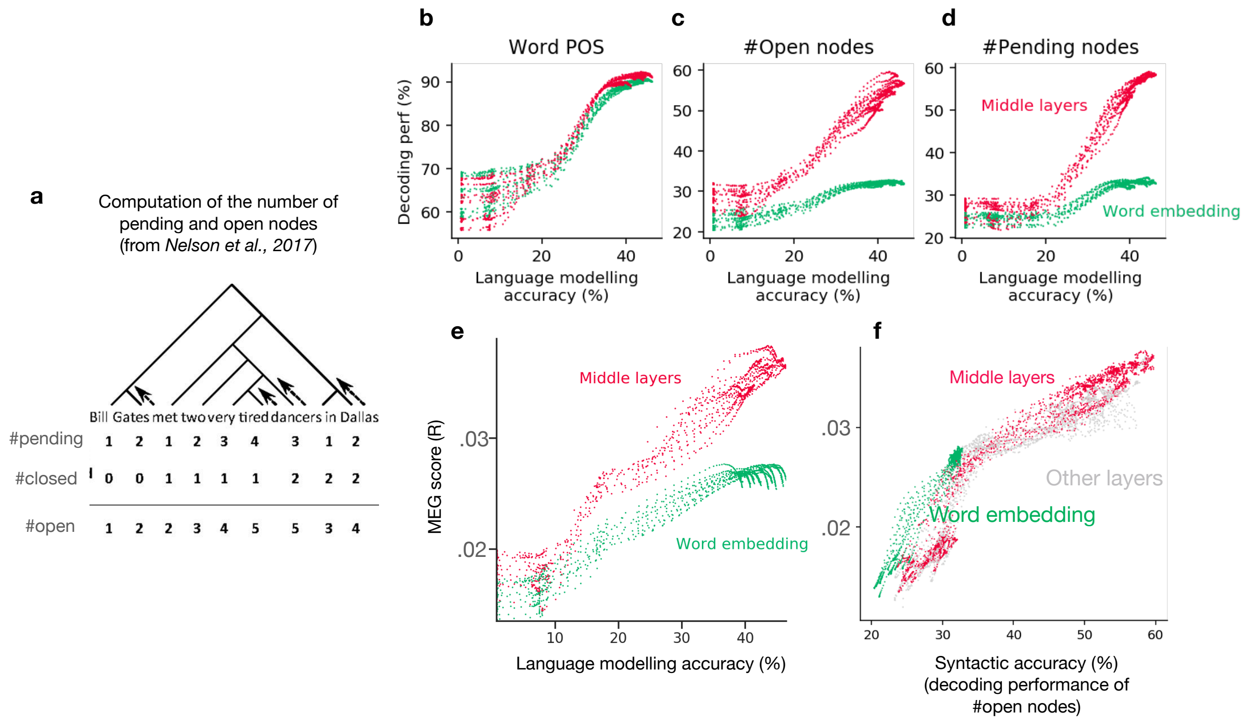

Supplementary Figure 2: **What linguistic information drives the brain score? a.** From the stimulus, we compute three linguistic features: the part-of-speech of the words (i) (as given by Spacy), and two higher-level syntactic features: the number of pending nodes (ii) and open nodes (iii). These two syntactic features are derived from the constituency trees of the sentences, following [8]. **b-d**. A $\ell 2$-penalized linear regression is fit to predict the three linguistic features from the word embeddings (green), and middle layers (red) of the causal models studied in Figure 4b. The decoding performance is reported on the y-axis (accuracy at predicting the part-of-speech for b, r-squared for c, d and e). **e.** MEG scores (averaged across sensors and time) of the embeddings given their language modeling performance (top-1 accuracy at predicting the next word, Figure 4b). **f.** MEG scores of the embeddings given their ability to predict the number of open nodes.

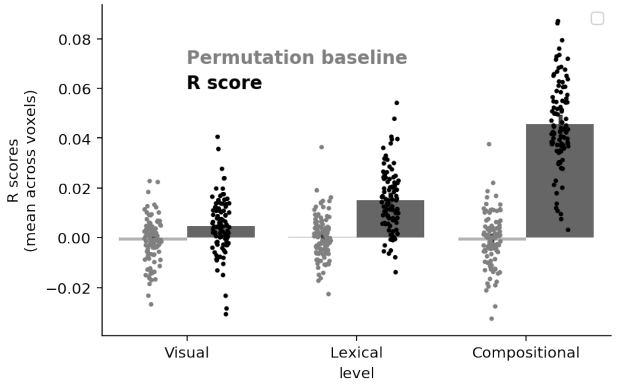

Supplementary Figure 3: **Permutation distribution.** As a baseline, we compare the normal R scores (dark colors) to those of a permutation distribution (light colors) for each of the visual, lexical and compositional embeddings introduced in Figure 3. For each (subject, voxel) pair, we compute the mapping between the embeddings X and the fMRI of the subject, either (i) shuffled across time samples or (ii) without shuffling. Above, we report scores averaged across subjects and voxels. Error bars are standard-error of the mean across subjects (n=100).

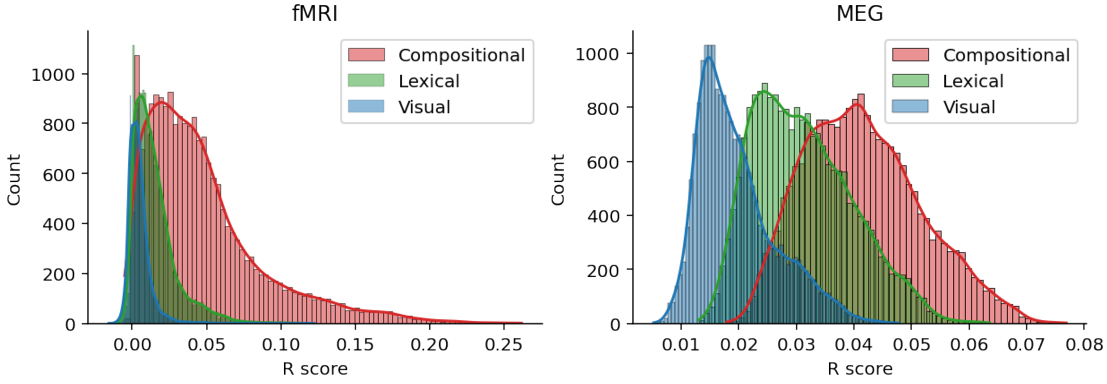

Supplementary Figure 4: **Distribution of R scores across fMRI voxels (left) and MEG sources (right).** We compute the brain scores for the visual (blue), lexical (green) and compositional (red) embeddings introduced in Figure 3. We average scores across voxels (resp. sources) and subjects, to obtain one single score per voxel (resp. source). Above, the corresponding distribution of the R scores across voxels and sources.

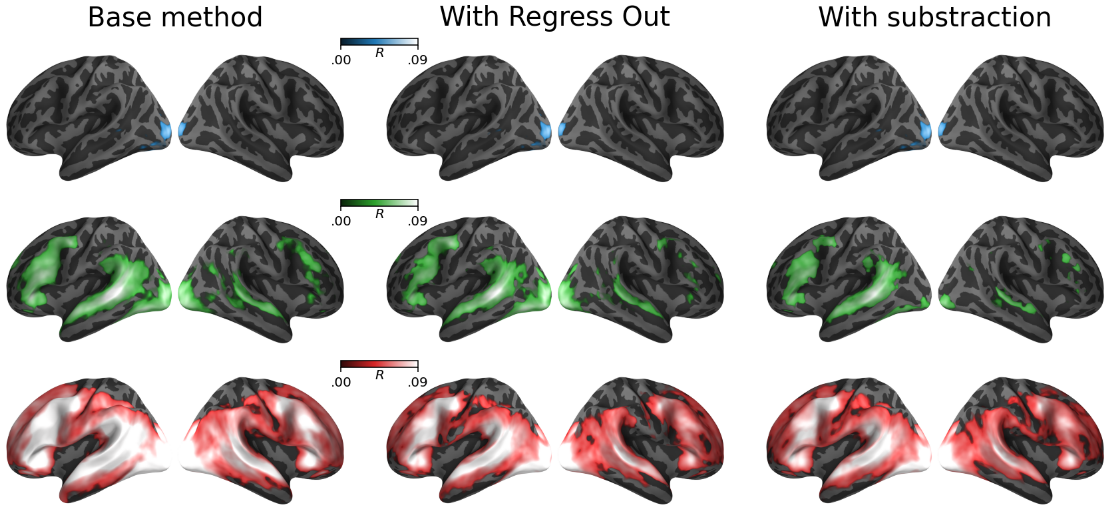

Supplementary Figure 5: **Comparison between two orthogonalization methods.** In Figure 3, we report the raw brain scores (without subtraction) for the visual (blue, X_V), lexical (green, X_W) and compositional (red, X_C) embeddings ("base method" on the left). On the right, for each level, we subtract the scores of the level below (e.g. red scores $R_C = \mathcal{R}(X_C) - \mathcal{R}(X_W)$). In the middle, we orthogonalize the predictors before computing the brain scores, by"regressing ou" the effect of the lower level onto the current level. For the compositional score R_C, we fit a ridge regression model f (we use the RidgeCV implementation from scikit-learn, with 10 possible penalization values log spaced between 10^{-3} and 10^8) to predict X_C given the concatenation of the visual and word embeddings $X_V \oplus X_W$. Then, we compute the brain scores of the residuals $\tilde{X_C} = X_C - \hat{f}(X_V \oplus X_W)$. We proceed similarly for the lexical residuals $\tilde{X}_W = X_W \ X_V$. As we see, the subtraction method (right) is more conservative than the method with regress out (middle).

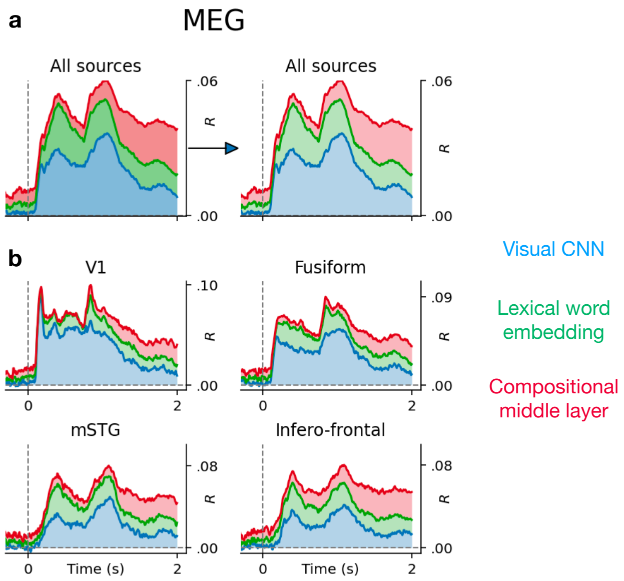

Supplementary Figure 6: **Brain scores over time. a)** Same as Figure 3c, but without subtracting the scores of the level below. **b)** Same as Figure 3c without subtracting the scores.

Fronto-polar cortex:	0.054 ± 0.003	$p < 10^{-8}$
Fusiform:	0.120 ± 0.004	$p < 10^{-8}$
Infero-frontal:	0.139 ± 0.005	$p < 10^{-8}$
M1:	0.042 ± 0.003	$p < 10^{-8}$
STG:	0.129 ± 0.004	$p < 10^{-8}$
Supramarginal:	0.078 ± 0.003	$p < 10^{-8}$
V1:	0.150 ± 0.006	$p < 10^{-8}$

Supplementary Table 1: **Average noise ceiling within each region-of-interest.** Mean, standard error of the mean and p-values across subjects.

Task	Dim	Layers	Heads	Best perplexity	Best accuracy
mlm	512	12	8	4.70	67.51
mlm	512	12	4	4.70	67.36
mlm	512	8	4	4.90	66.72
mlm	512	8	8	4.99	66.33
mlm	512	4	8	5.55	64.40
mlm	512	4	4	5.90	63.61
mlm	256	12	8	6.08	63.48
mlm	256	12	4	6.12	63.36
mlm	256	8	8	6.62	62.12
mlm	256	8	4	6.69	61.71
mlm	256	4	8	7.75	59.73
mlm	256	4	4	7.97	59.15
mlm	128	12	8	8.99	57.65
mlm	128	12	4	9.26	57.46
mlm	128	8	8	10.01	56.35
mlm	128	8	4	10.11	56.16
mlm	128	4	8	12.06	53.70
mlm	128	4	4	12.60	53.08
clm	512	12	8	15.00	46.47
clm	512	12	4	15.06	46.38
clm	512	8	4	15.49	46.01
clm	512	8	8	15.49	45.97
clm	512	4	8	16.75	44.93
clm	512	4	4	16.90	44.82
clm	256	12	4	17.85	44.28
clm	256	12	8	17.80	44.26
clm	256	8	8	18.69	43.68
clm	256	8	4	18.83	43.59
clm	256	4	4	20.67	42.53
clm	256	4	8	20.64	42.49
clm	128	12	4	23.26	41.47
clm	128	12	8	23.31	41.38
clm	128	8	4	24.45	40.83
clm	128	8	8	24.36	40.80
clm	128	4	4	27.11	39.61
clm	128	4	8	27.06	39.57

Supplementary Table 2: **Performance of the 36 transformer architectures.** Best perplexity (the lower the better) and top-1 accuracy (the higher the better) of 36 transformer architectures, evaluated on a test test of $\approx$180K words from Wikipedia. Transformers are trained with a masked ('mlm') or causal ('clm') language modeling objective. They vary in their dimensionality ('Dim'), number of layers ('Layers') and number of attention heads ('Heads'). The models are trained on a set of $\approx$280K words from Wikipedia (in Dutch). The training is stopped when the perplexity on a validation set does not decrease for 5 epochs.

Supplementary References

[1] Charlotte Caucheteux, Alexandre Gramfort, and Jean-Rémi King. "Model-based analysis of brain activity reveals the hierarchy of language in 305 subjects". In: *EMNLP 2021 - Conference on Empirical Methods in Natural Language Processing*. Punta Cana (and Online), Dominican Republic, Nov. 2021. (Visited on 10/14/2021).

[2] Charlotte Caucheteux, Alexandre Gramfort, and Jean-Remi King. "Disentangling Syntax and Semantics in the Brain with Deep Networks". In: *arXiv:2103.01620 [cs, q-bio]* (June 2021). arXiv: 2103.01620. (Visited on 09/28/2021).

[3] Stanislas Dehaene and Laurent Cohen. "The unique role of the visual word form area in reading". In: *Trends in cognitive sciences* 15.6 (2011), pp. 254–262.

[4] Evelina Fedorenko et al. "Lack of selectivity for syntax relative to word meanings throughout the language network". In: *bioRxiv* (2020), p. 477851.

[5] Evelina Fedorenko et al. "Neural correlate of the construction of sentence meaning". In: *Proceedings of the National Academy of Sciences* 113.41 (2016), E6256–E6262.

[6] Peter Hagoort. "The neurobiology of language beyond single-word processing". In: *Science* 366.6461 (2019), pp. 55–58.

[7] Gregory Hickok and David Poeppel. "The cortical organization of speech processing". In: *Nature Reviews Neuroscience* 8.5 (May 2007). Number: 5 Publisher: Nature Publishing Group, pp. 393–402. ISSN: 1471-0048. DOI: 10.1038/nrn2113. (Visited on 06/11/2020).

[8] Matthew J. Nelson et al. "Neurophysiological dynamics of phrase-structure building during sentence processing". en. In: *Proceedings of the National Academy of Sciences* 114.18 (May 2017). Publisher: National Academy of Sciences Section: PNAS Plus, E3669–E3678. ISSN: 0027-8424, 1091-6490. DOI: 10.1073/pnas.1701590114. (Visited on 04/14/2021).

[9] Francis Jeffry Pelletier. "The principle of semantic compositionality". In: *Topoi* 13.1 (1994), pp. 11–24.

[10] Zoltán Gendler Szabó. "Compositionality". In: (2004).
