# Supplementary material for: Brains and algorithms partially converge in natural language processing
Source: Commun Biol. 2022 Feb 16;5:134. doi: 10.1038/s42003-022-03036-1 (PMC8850612; doi:10.1038/s42003-022-03036-1)
Supplement: Supplementary file 3 — Description of Additional Supplementary Files [file 42003_2022_3036_MOESM3_ESM.pdf]

Description of Additional Supplementary Files

File Name: Supplementary Movie 1
Description: Anatomical and temporal hierarchy of reading. Average brain responses elicited by the onset of visual words (~2,700 words were presented to each of the 95 subjects), as estimated with minimum source estimates (MNE) of the single-trial responses constrained by the individual subjects' anatomy (cortical surface extracted from T1 scans). These results correspond to the ones summarized in Figure 1A. Overall, these results confirm that we can track the sequential recruitment of the cortical hierarchy of reading, starting from early visual cortex, moving up through the expected location of the visual word form area, and then igniting the temporal, prefrontal and parietal areas typically associated with language processing. Although these effects are bilateral, the typical left-lateralization associated with language processing can be observed.

File Name: Supplementary Movie 2
Description: The main levels of the hierarchy of language revealed by deep neural networks. Single-trial encoding scores obtained for three representative embeddings reveal the types of representations that are generated within each region and at each instant. Blue, green and red colors indicate when and Page 7 of 23 where brain responses to words are specifically predicted by visual, word and contextual embeddings, respectively (a.k.a gain in brain scores). The animated legend illustrates the same data without the anatomy: each dot corresponds to a brain source, radius corresponds to effect size (center: no effect, circle: maximum effect), and angle corresponds to the type of representation (visual, lexical or contextual). Overall, these results show when and where the brain transforms visual representations into lexical and contextual representations.

File Name: Supplementary Data 1
Description: Data used to generate Figure 2.
a) Average MEG response for each MEG source and time step (Figure 2a).
b) Average noise ceiling estimates for each MEG source and time step (Figure 2b).
c) Average noise ceiling estimate for each MEG channel and time step (Figure 2c).
d) Average noise ceiling estimate for each fMRI voxel (Figure 2d).

File Name: Supplementary Data 2
Description: Data used to generate Figure 3.
a) Brain scores for the visual, lexical and compositional embeddings, for each subject and fMRI voxel (Figure 3a).
b) Brain scores for the visual, lexical and compositional embeddings, for MEG source and time step (Figure 3bcd).

File Name: Supplementary Data 3
Description: Data used to generate Figure 4.
a) Brain scores (MEG) for each subject, for each layer of six representative language transformer models (all 128 units per layer, eight heads) trained with a causal (clm) or masked (mlm) language modelling task (Figure 4a).
b) Brain scores (MEG) over training, average across subjects and channels, for 16 causal transformer architectures (Figure 4b).
c) Permutation importance (MEG) for each subject and property (accuracy, layer depth or position, training task, dimensionality, number of heads and number of layers, Figure 4d).
d) Brain scores (fMRI) for each subject, for each layer of six representative language transformer model (all 128 units per layer, eight heads) trained with a causal (clm) or masked (mlm) language modelling task (Figure 4e).
e) Brain scores (fMRI) over training, average across subjects and channels, for 16 causal transformer architectures (Figure 4f).
f) Permutation importance (fMRI) for each subject and property (accuracy, layer depth or position, training task, dimensionality, number of heads and number of layers, Figure 4h).
