# Supplementary material for: Brains and algorithms partially converge in natural language processing
Source: Commun Biol. 2022 Feb 16;5:134. doi: 10.1038/s42003-022-03036-1 (PMC8850612; doi:10.1038/s42003-022-03036-1)
Supplement: Supplementary file 9 — Reporting Summary [file 42003_2022_3036_MOESM9_ESM.pdf]

nature portfolio

Corresponding author(s): Charlotte Caucheteux

Last updated by author(s): Nov 25, 2021

Reporting Summary

Nature Portfolio wishes to improve the reproducibility of the work that we publish. This form provides structure for consistency and transparency in reporting. For further information on Nature Portfolio policies, see our Editorial Policies and the Editorial Policy Checklist.

Statistics

For all statistical analyses, confirm that the following items are present in the figure legend, table legend, main text, or Methods section.

n/a	Confirmed	
☐	☒	The exact sample size (n) for each experimental group/condition, given as a discrete number and unit of measurement
☐	☒	A statement on whether measurements were taken from distinct samples or whether the same sample was measured repeatedly
☐	☒	The statistical test(s) used AND whether they are one- or two-sided<br>*Only common tests should be described solely by name; describe more complex techniques in the Methods section.*
☐	☒	A description of all covariates tested
☐	☒	A description of any assumptions or corrections, such as tests of normality and adjustment for multiple comparisons
☐	☒	A full description of the statistical parameters including central tendency (e.g. means) or other basic estimates (e.g. regression coefficient) AND variation (e.g. standard deviation) or associated estimates of uncertainty (e.g. confidence intervals)
☐	☒	For null hypothesis testing, the test statistic (e.g. F, t, r) with confidence intervals, effect sizes, degrees of freedom and P value noted<br>*Give P values as exact values whenever suitable.*
☒	☐	For Bayesian analysis, information on the choice of priors and Markov chain Monte Carlo settings
☐	☒	For hierarchical and complex designs, identification of the appropriate level for tests and full reporting of outcomes
☐	☒	Estimates of effect sizes (e.g. Cohen's d, Pearson's r), indicating how they were calculated

Our web collection on statistics for biologists contains articles on many of the points above.

Software and code

Policy information about availability of computer code

Data collection	No data collection was performed.
Data analysis	Data analysis was performed in Python using the scikit-learn open source library. The MEG and fMRI data were processed using MNE-Python and freesurfer. The natural language processing algorithms were trained using the implementation from the XLM github repository (https://github.com/facebookresearch/XLM).

For manuscripts utilizing custom algorithms or software that are central to the research but not yet described in published literature, software must be made available to editors and reviewers. We strongly encourage code deposition in a community repository (e.g. GitHub). See the Nature Portfolio guidelines for submitting code & software for further information.

Data

Policy information about availability of data

All manuscripts must include a data availability statement. This statement should provide the following information, where applicable:

- Accession codes, unique identifiers, or web links for publicly available datasets
- A description of any restrictions on data availability
- For clinical datasets or third party data, please ensure that the statement adheres to our policy

The data are publicly available on request. They were provided by the Donders Institute for Brain, Cognition and Behaviour after having been approved by the local ethics committee (CMO – the local "Committee on Research Involving Human Subjects" in the Arnhem-Nijmegen region). Link: https://data.donders.ru.nl/collections/di/dccn/DSC_3011020.09_236?5

Field-specific reporting

Please select the one below that is the best fit for your research. If you are not sure, read the appropriate sections before making your selection.

☒ Life sciences ☐ Behavioural & social sciences ☐ Ecological, evolutionary & environmental sciences

For a reference copy of the document with all sections, see nature.com/documents/nr-reporting-summary-flat.pdf

Life sciences study design

All studies must disclose on these points even when the disclosure is negative.

Sample size	102
Data exclusions	Two subjects were excluded from the fMRI analyses because of difficulties processing the metadata, resulting in 100 fMRI subjects. Seven subjects were excluded from the MEG analyses because of difficulties processing the metadata,resulting in 92 MEG subjects.
Replication	The results were validated on two modalities (MEG and fMRI) and 3,600 neural networks.
Randomization	For each subject, the stimulus (sentences) was split into five folds. Then, a model was trained on four folds (75% of the words) and tested on the last fold (25% of held out words). The procedure was repeated for each possible split (five) and the results were averaged across the five test folds.
Blinding	No blinding was performed because one model was trained independently for each subject.

Reporting for specific materials, systems and methods

We require information from authors about some types of materials, experimental systems and methods used in many studies. Here, indicate whether each material, system or method listed is relevant to your study. If you are not sure if a list item applies to your research, read the appropriate section before selecting a response.

Materials & experimental systems

n/a	Involved in the study	
☒	☐	Antibodies
☒	☐	Eukaryotic cell lines
☒	☐	Palaeontology and archaeology
☒	☐	Animals and other organisms
☒	☐	Human research participants
☒	☐	Clinical data
☒	☐	Dual use research of concern

Methods

n/a	Involved in the study	
☒	☐	ChIP-seq
☒	☐	Flow cytometry
☒	☐	MRI-based neuroimaging
